# Early and eighteen month clinical outcomes of first UK case of percutaneous deep vein arterialisation (pDVA) to treat “no option” chronic limb-threatening ischemia using the LimFlow system

**DOI:** 10.1186/s42155-021-00252-4

**Published:** 2021-08-09

**Authors:** Symeon Lechareas, Kaji Sritharan, R. G. Mc Williams

**Affiliations:** 1 Radiology department Royal Liverpool Hospital, Liverpool, England; 2 Department of Vascular Surgery, Royal Liverpool Hospital, Liverpool, England

**Keywords:** Chronic limb-threatening ischaemia, Percutaneous deep vein arterialisation, Amputation, No-option chronic limb-threatening ischaemia

## Abstract

**Background:**

Chronic limb-threatening ischaemia (CLTI) in cases where there are no further standard treatment options for limb salvage represents the most advanced stage of peripheral arterial disease. For these “no-option” CLTI patients, an experimental treatment of foot vein arterialisation (FVA) was first described in 1912, however, it was never widely adopted as outcomes varied significantly most likely due to the complexity of the surgical intervention and lack of standardisation. In recent years there have been significant developments in performing FVA fully percutaneously and standardising the procedure with the introduction of specific indications for patient selection, a dedicated set of devices and structured follow up. This case represents the first UK use of the dedicated LimFlow System as a standardised procedure to perform percutaneous deep vein arterialisation (pDVA) in a “no option” CLTI patient according to the latest treatment recommendations in the literature, with outcomes out to 18 months post-procedure.

**Case presentation:**

We present the case of a 78 year old male diabetic patient with a history of contralateral below knee amputation who presented with ischaemic rest pain and dry gangrene involving his left heel and first and second toes. Following review by the lower limb multi-disciplinary team at our institution, the patient was deemed to have no surgical or endovascular treatment options, apart from major amputation, as there was no suitable target for either angioplasty or bypass. He was therefore referred as a candidate for percutaneous deep vein arterialisation (pDVA) with the LimFlow System (LimFlow SA, France). After screening of the patient according to the indications for use, the pDVA procedure was successfully performed resulting in complete resolution of ischaemic rest pain immediately following the procedure, and adequate revascularisation of the foot. Following the index procedure, the subject went on to have minor amputation of the first, second and third toes 2 months post initial procedure with further secondary angioplasty procedures to optimise the flow throughout the arterialised circuit up to 4 months after the initial procedure. He underwent elective completion transmetatarsal amputation at 13 months post index procedure. The surgical wounds post minor amputation and the heel wound showed continued healing, especially after secondary optimisation of the pDVA outflow, with tissue epithelialisation by 6 months and complete healing by 18 months after the index procedure.

**Conclusions:**

This case report demonstrates the clinical outcomes of a technically-successful standardised pDVA procedure with the LimFlow system including both limb salvage and wound healing at 18 months. It also highlights the importance of close clinical and radiological surveillance post-index procedure and the requirement for re-interventions to optimise wound healing.

## Background

Chronic limb-threatening ischaemia (CLTI) originates in occlusive arterial disease and is characterised by ischaemic rest pain and foot wounds including ulcers or gangrene classified as Rutherford class 4–6. On its own, CLTI has a prognosis of 20% mortality at 6 months and over 50% at 5 years (Adam et al., 2005; Stoyioglou & Jaff, 2004; Abu Dabrh et al., 2015). Amongst these patients, 20% have no further options for surgical or endovascular treatment due to the lack of suitable distal target vessel or due to other co-morbities (Faglia et al., 2009). These so called “no-option” CLTI patients have an even worse prognosis of 40% major amputation and 20% mortality at 6 months (Norgren et al., 2007). The lack of options for treating these patients has motivated physicians to try the concept of vein arterialisation or arteriovenous anastomosis for the purpose of limb salvage from as early as 1912 (Halstead & Vaughan, 1912). However, several aspects of the applied technique, including patient selection, anatomical location of arteriovenous anastomosis and method of treating competent vein valves in the distal foot, varied considerably which likely caused the inconsistent results reported with venous arterialisation procedures (Schreve et al., 2017). The introduction of a standardised percutaneous technique for deep vein arterialisation (pDVA) with dedicated devices, specific indications for use and defined follow-up protocols, has allowed more consistent results. A multi-centre study of 32 patients recorded amputation free survival (AFS) of 84% and 71% at 6 and 12-months respectively where pDVA has been performed using the dedicated LimFlow System. Further to avoiding amputation, the re-establishment of blood flow to the foot after pDVA ultimately allows complete wound healing in 37% and 68% at 6 and 12-months respectively of patients treated with this procedure, and when follow-up treatment is applied in a manner that optimises outcomes (Schmidt et al., 2020).The exact effects of the pDVA in the circulation of the ischemic foot and the mechanisms behind limb salvage are still under investigation. It has been speculated that reversal of flow all the way through the capillaries improves tissue nutrition and possibly stimulates angiogenesis (Schreve et al., 2019). In this case report we present the first experience in the UK of this standardised approach for the pDVA procedure with the LimFlow system and describe the process of patient selection, procedure planning and appropriate follow up to ensure successful limb salvage and wound healing. Patient selection criteria, as indicated for use with this device, include a clinical diagnosis of symptomatic chronic critical limb ischemia, patients have been assessed by a vascular surgeon and interventionalist who have determined that no surgical or endovascular treatment is possible, and are clearly indicated for major amputation (so-called “no-option” patients). The inclusion and exclusion criteria have been described in detail in the study protocol of PROMISE International trial (Schreve et al., 2019) .Main exclusion criteria for this treatment are: acute limb ischemia, deep venous thrombosis (DVT) and poor cardiac function.

## Case report

**Fig. 1:**
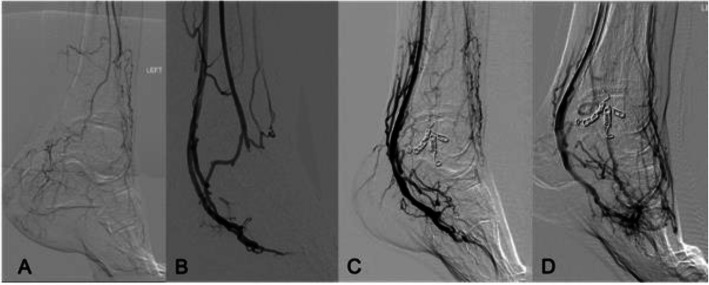
**A** Baseline angiogram, 1 month prior to pDVA **B**) post index pDVA, **C**) post collateral vein embolization **D**) post final outflow optimisation with angioplasty of the pedal venous arch)

A 78-year old male patient with recent contralateral right below-the-knee amputation initially presented in July 2019 with ischaemic rest pain and dry gangrene involving his left heel and his first and second toes. His comorbidities included type II diabetes, hypertension, previous stroke and hypercholesterolaemia. Duplex ultrasound showed a focal stenotic lesion in the distal SFA and heavily calcific occlusive tibial disease. He was classified as Rutherford category 5 with a Society for Vascular Surgery WIfI “wound, ischemia and foot infection” score of 2–3-0, which meant he was at high risk of major amputation within the year. The patient initially underwent balloon angioplasty of the SFA but angiography revealed poor outflow to the foot with no suitable target for distal angioplasty or bypass (Fig. 1A). The lower limb multidisciplinary team at our institution reviewed the case and agreed to proceed with pDVA as the only possible treatment apart from major amputation.

The patient underwent vein assessment prior to the procedure and this included duplex scan imaging of the superficial and deep venous system of the foot. Good calibre lateral plantar and posterior tibial veins are necessary for a successful outcome while the presence of DVT contraindicates the procedure. The arterial inflow was assessed in detail on the previous conventional angiograms and the crossing point was determined to be in the proximal posterior tibial artery. The peroneal artery was avoided as a potential ‘crossing’ artery since it supplied the ischemic foot with useful collaterals. Active foot infection, significant cardiac history were excluded and life-expectancy and functional status were also considered before listing the patient for intervention.

The pDVA procedure was performed in August 2019 as the first use of the LimFlow System in the United Kingdom. Under ultrasound guidance, arterial access was performed antegrade in the left CFA and venous access was performed in the lateral plantar vein. Using the LimFlow arterial and venous catheters, an arterial-venous communication was created at the level of the proximal posterior tibial artery into a posterior tibial vein. The target posterior tibial and lateral plantar veins were then treated with the LimFlow antegrade (push) valvulotome to render the valves incompetent which was confirmed with balloon dilatation. Finally, the LimFlow self-expanding PTFE-covered stents were deployed in the posterior tibial vein distally from the ankle level to the crossing location where a conical stentgraft was deployed to secure the fistula and optimise flow transition from the artery into the vein. The completed pDVA circuit successfully established blood-flow to the foot (Fig. 1B) and post-procedure ultrasound demonstrated a satisfactory volume flow of 250 cc/min in the arterialised lateral plantar vein. The case took just under three hours from the time of initial access until final access site closure.

**Fig. 2:**
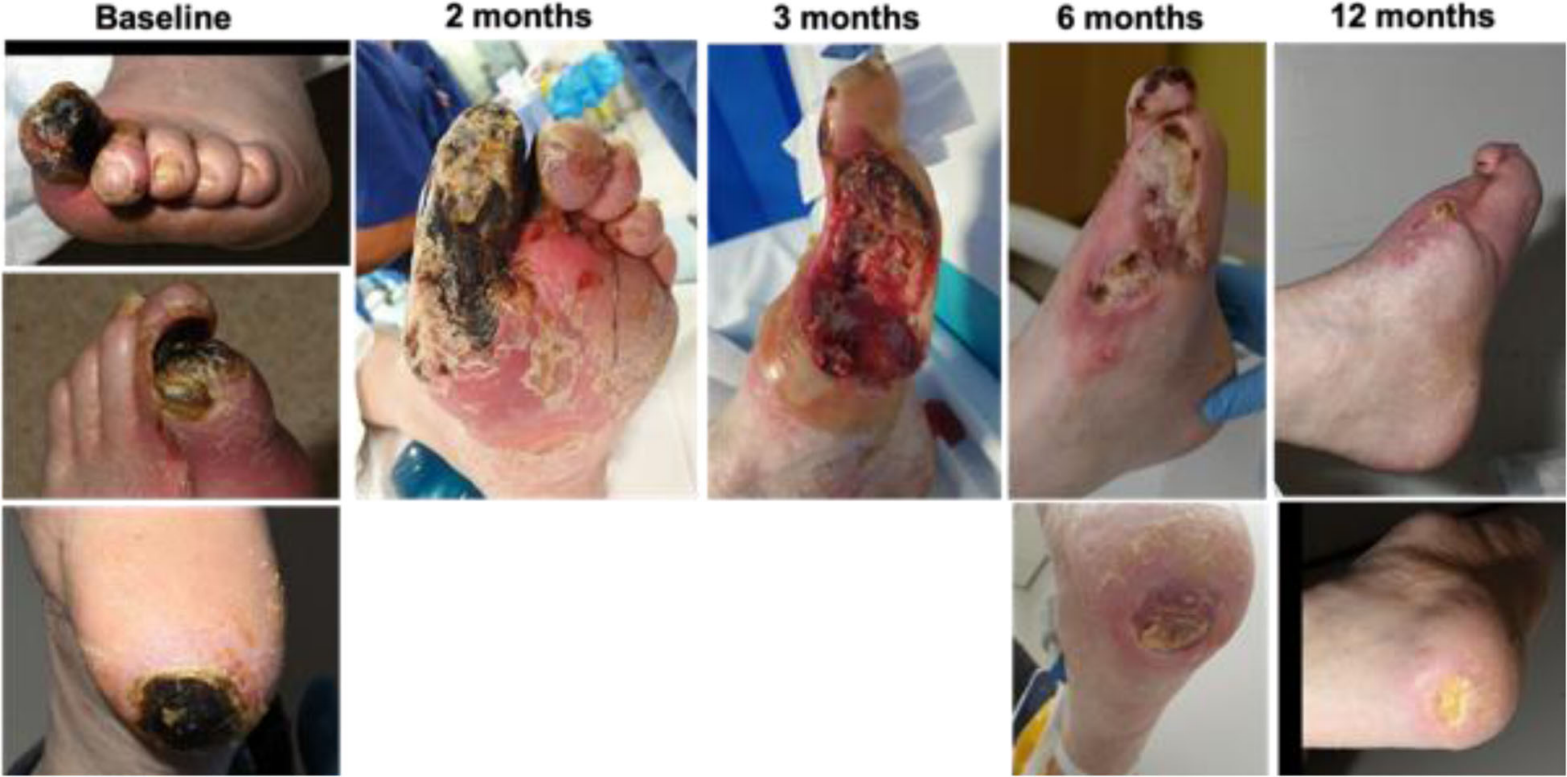
Progression of wound healing baseline to 12 months post pDVA)

**Fig. 3:**
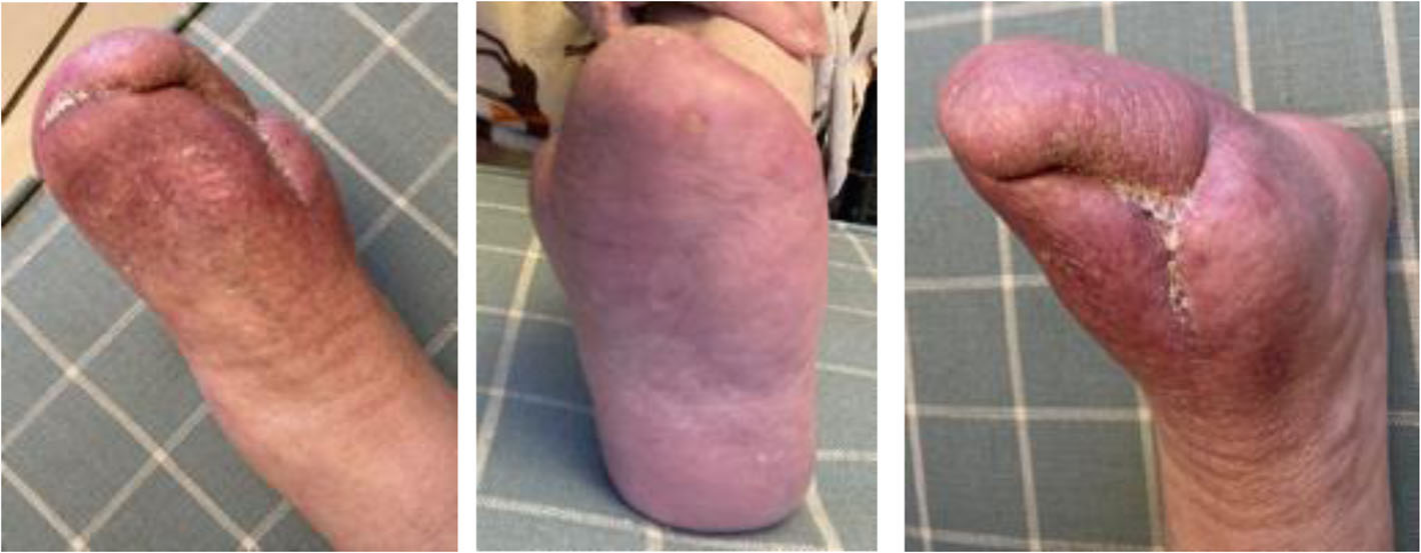
Most recent images of the foot, 18 months post pDVA)

The procedure was technically successful with no perioperative complications. The patient experienced an almost complete resolution of ischaemic rest pain after the procedure and did not experience any adverse events in the first 60 days. He was discharged home at day 18 post-procedure to the care of the community diabetic foot and district nursing teams to oversee his foot wound care. Ten weeks after his index procedure, the patient presented to the emergency department with diabetic foot sepsis most likely due to a combination of superimposed infection of the existing necrotic tissue and ischaemia. He underwent first, second and third toe amputation and foot debridement for sepsis control. During his admission to hospital, the patient also had a duplex ultrasound to monitor flow into the affected area, his third surveillance scan as part of a defined follow up protocol to closely monitor and optimise blood flow as necessary. Consequently, angioplasty was performed at the same sitting as surgical debridement to treat the narrowing of the inflow and outflow tracts of the stent grafts at the level of the origin of the posterior tibial artery as well as in the lateral plantar vein. No signs of in-stent stenosis were observed at the time of inflow and outflow treatment. Further surveillance duplex imaging at 3 months revealed a reduction of flow in the stent due to narrowing in the lateral plantar vein. Although there had been no clinical deterioration, further intervention was performed with the implantation of a Supera stent into the lateral plantar vein distal to the LimFlow stent in order to enhance the flow in the venous arch. This was followed by coil embolisation of collateral veinsin order to focalise blood flow to the distal foot. Stealing branches of the great saphenous vein were embolised in the foot in order to encourage flow in the deep venous system and consequently improve healing. At 4 months, the patient underwent a final maintenance angioplasty where the outflow pedal venous arch was treated with balloon angioplasty to further aid circulation to the area of the toe amputation (Fig. 1D). The heel ulcer and minor amputation wounds showed continued improvement post-pDVA with tissue granulation after debridement at 2 months and complete epithelialisation by 12 months (Fig. 2). Ischaemic rest pain resolved almost completely after the index procedure and the patient did not report any pain by 6 months and onward. Due to bone protrusion from the wound, a completion transmetatarsal amputation was performed at 13 months. Most recent Images of the foot (Fig. 3) obtained 18 months post index procedure, showing complete wound healing and preservation of the foot.

## Conclusions

In conclusion, this first UK case of the LimFlow System in treating a no-option CLTI patient with pDVA demonstrates the feasibility of this treatment in terms of safety and effectiveness, not only acutely with the creation of blood flow to the extremity as evidenced by a resolution of ischaemic rest pain, but also with respect to the longer term clinical outcomes of limb preservation and wound healing. Success in this case rested on the careful selection of a patient with no-option CLTI, alongside a follow-up protocol designed to carefully monitor blood flow to the affected area using duplex ultrasound, and angioplasty treatment where indicated from those results. Endovascular and surgical reinterventions are common in order to optimise flow and wound healing. Although promising, the exact mechanism of action of the pDVA is still speculated and under investigation and therefore more studies are needed to assess the short and long term outcomes of the technique. The successful clinical outcome of the first commercial pDVA in the UK performed in our department motivated our team to participate in the enrolment of the PROMISE UK trial which is an ongoing multicenter national trial.

## Data Availability

Not applicable.

## Abbreviations

CLTI: Chronic limb-threatening ischaemia
pDVA: Percutaneous deep vein arterialisation
DVT: Deep Venous thrombosis
